# Anti-Apoptotic Role of Sanhuang Xiexin Decoction and Anisodamine in Endotoxemia

**DOI:** 10.3389/fphar.2021.531325

**Published:** 2021-04-21

**Authors:** Zixuan Liu, Wenxiang Wang, Jie Luo, Yingrui Zhang, Yunsen Zhang, Zhiqiang Gan, Xiaofei Shen, Yi Zhang, Xianli Meng

**Affiliations:** ^1^ Ethnic Medicine Academic Heritage Innovation Research Center, Chengdu University of Traditional Chinese Medicine, Chengdu, China; ^2^ School of Ethnic Medicine, Chengdu University of Traditional Chinese Medicine, Chengdu, China; ^3^ TCM Regulating Metabolic Diseases Key Laboratory of Sichuan Province, Hospital of Chengdu University of Traditional Chinese Medicine, Chengdu, China; ^4^ Innovative Institutes of Chinese Medicine and Pharmacy, Chengdu University of Traditional Chinese Medicine, Chengdu, China

**Keywords:** endotoxemia, anti-apoptosis, immunosuppression, herbal medicine, sanhuang xiexin decoction, anisodamine

## Abstract

Endotoxemia is characterized by initial uncontrollable inflammation, terminal immune paralysis, significant cell apoptosis and tissue injury, which can aggravate or induce multiple diseases and become one of the complications of many diseases. Therefore, anti-inflammatory and anti-apoptotic therapy is a valuable strategy for the treatment of endotoxemia-induced tissue injury. Traditional Chinese medicine exhibits great advantages in the treatment of endotoxemia. In this review, we have analyzed and summarized the active ingredients and their metabolites of Sanhuang Xiexin Decoction, a famous formula in endotoxemia therapy. We then have summarized the mechanisms of Sanhuang Xiexin Decoction against endotoxemia and its mediated tissue injury. Furthermore, silico strategy was used to evaluate the anti-apoptotic mechanism of anisodamine, a well-known natural product that widely used to improve survival in patients with septic shock. Finally, we also have summarized other anti-apoptotic natural products as well as their therapeutic effects on endotoxemia and its mediated tissue injury.

**GRAPHICAL ABSTRACT:**
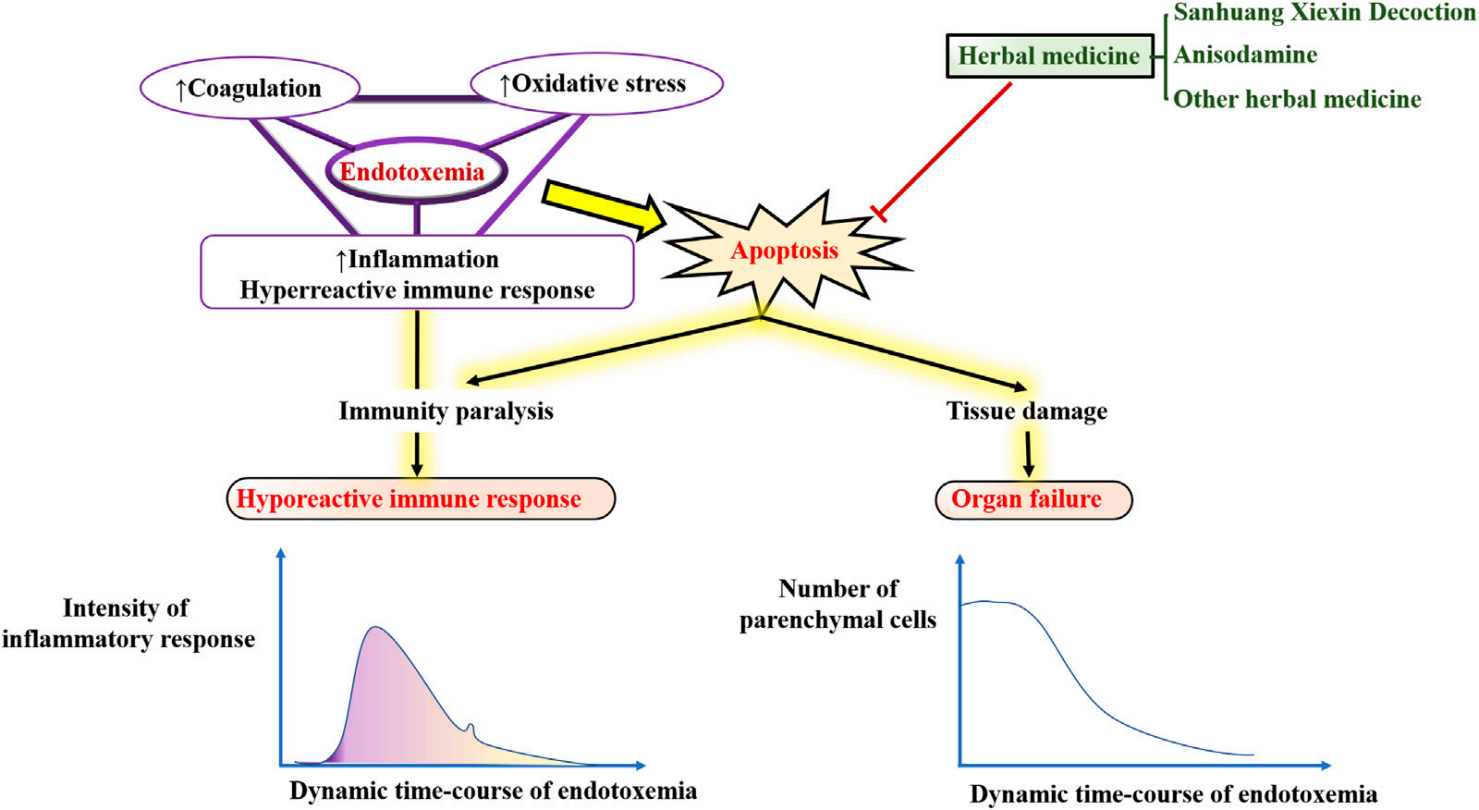


## Introduction

Possible sources of plasma endotoxin are bacteria from infected local tissues, blood, respiratory tract, digestive tract, food, or other ingested matter (Munford, 2016). The intestinal and other epithelial cells, such as those of the skin or lungs, are the first line of defense against endotoxins entering the bloodstream. Bacteria release bacterial lipopolysaccharide (LPS) or endotoxins mainly through lymphatic channels (e.g., small intestinal lymphatic system) and thoracic duct into the circulation from infected parts. Furthermore, LPS can also be absorbed from the gastrointestinal mucosa, especially the damaged intestinal mucosa (Brenchley, 2006) into the portal circulation. Erridge (Erridge, 2011) and Deitch (Deitch, 2012) showed that the largest source of LPS detected in the peripheral venous blood was from the small intestine. In addition, blood-borne bacteria can release LPS directly into the bloodstream to cause diseases, such as *N. meningitidis* (Ovstebo et al., 2005; Coureuil et al., 2014) and *E. coli* (Simpson et al., 2000). However, when the amount of endotoxin is overloaded, the host defense fails to eliminate and control the infection, leading to endotoxemia or even sepsis (Piwowar, 2014). In addition to endotoxemia/sepsis, excessive amounts of LPS are also implicated in the pathogenesis of a range of diseases, such as atherosclerosis, alcoholic liver disease, autoimmunity, metabolic syndrome, renal injury, multiple organ failure, depression, and chronic fatigue. Interestingly, these diseases both share a common pathophysiological basis: the imbalance between induction of apoptosis and resistance to apoptosis (Manco et al., 2010).

**FIGURE 1:**
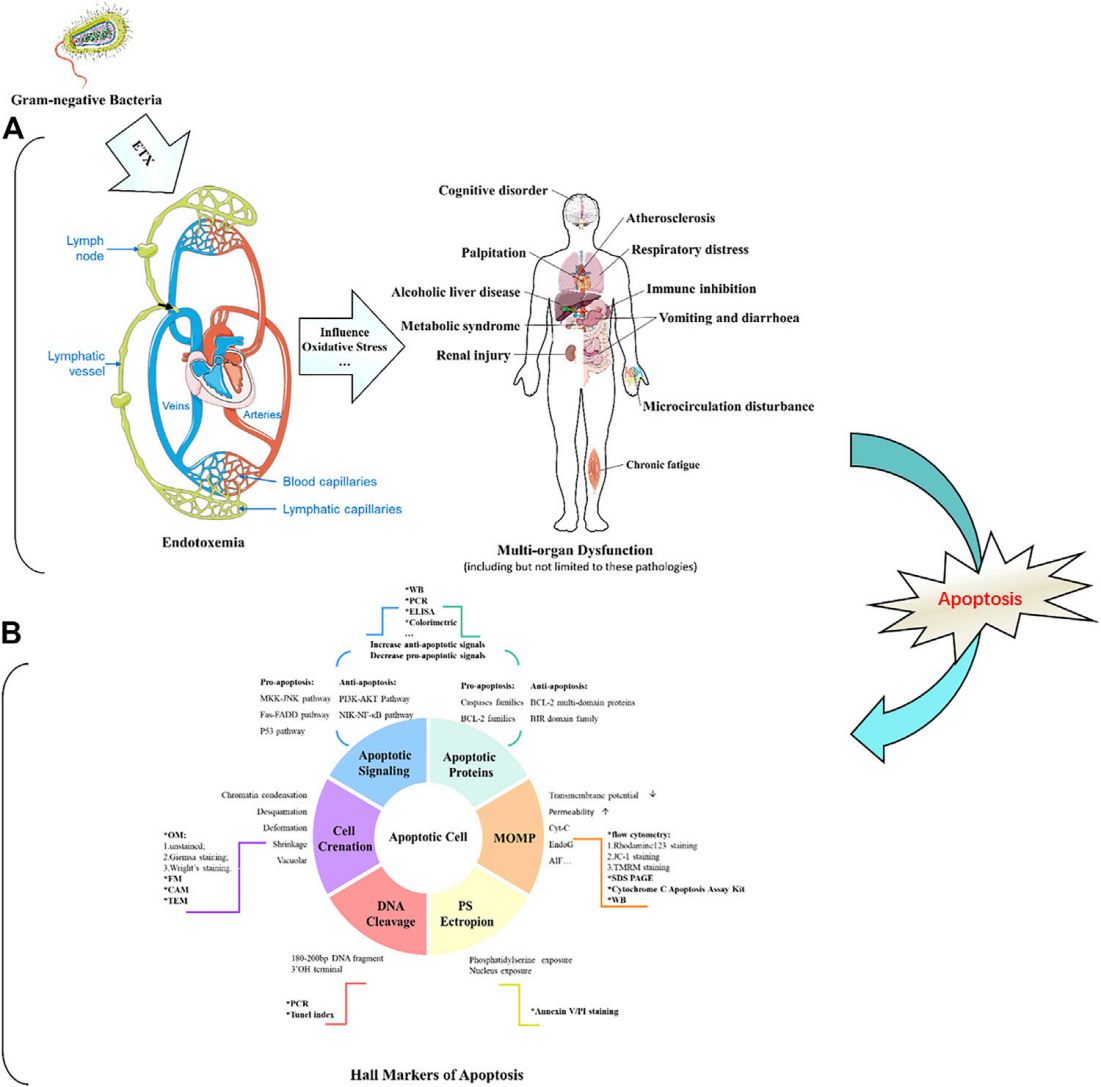
Schematic diagram of endotoxin damage and the detection of apoptotic cells.

Apoptosis is a conserved process of programmed cell death characterized by chromatin fragmentation and condensation, membrane blebbing, and nuclear collapse. Apoptosis also plays important roles in a variety of physiological processes, including embryogenesis, tissue remodeling, immune response, and carcinogenesis (Kerr et al., 1972; Williams, 1991). In this process, cells that are no longer needed or that will be detrimental to an organism or tissue are disposed of in a highly ordered manner. This process can effectively block the development of inflammatory responses and tumorigenesis. Figure 1 illustrates how endotoxemia occurs, with six apoptosis hallmarks and related assays to distinguish apoptosis cells from normal healthy ones.

It has been shown that various herbal ingredients exhibit potent anti-apoptotic effects and improve the survival rate in experimental endotoxemia models. In the last decade, an increasing number of studies have emphasized that the reduced incidence of various endotoxin-related pathologies is closely related to herbal medicine, which is a good source of beneficial natural anti-inflammatory, anti-oxidative stress, and anti-coagulant bioactive compounds. Moreover, the active ingredients of many herbs have been used to treat endotoxemia and its complications with impressive efficacy. For example, the administration of anisodamine significantly improved the survival rate of septic shock. Considering the complexity of the apoptosis-related endotoxemia mechanisms and the fact that the modulatory effects of herbs are difficult to be systematically presented in various experimental studies. Therefore, we wrote this review to provide a general and descriptive overview of the potential role and underlying mechanisms of apoptosis-related progression in endotoxemia, as well as biomarkers and assays for endotoxemia, and the bidirectional regulation of apoptosis in endotoxemia by herbal components and their metabolism (especially Sanhuang Xiexin decoction and Anisodamine).

This review explores these issues by taking a novel perspective on apoptosis regulation in the treatment of endotoxemia. It begins with the herbal ingredients that may affect the inflammatory response, visceral protection, and apoptosis of endotoxemia *in vitro* and *in vivo*. How the major herbal ingredients are metabolized will then be discussed, followed by a summary of these metabolites and their effects. The review concludes by considering the influencing factors that determine whether herbal ingredients regulating apoptosis are advantageous in therapeutic endotoxemia.

## Endotoxemia Is Associated With Various Diseases

Numerous variables can be activated in endotoxemia, including circulating glucocorticoid, cytotoxic T lymphocytes, oxygen free radicals, nitric oxide, heat shock proteins, FAS ligand, and pro-inflammatory cytokines (e.g., TNF-α, IL-1, and IL-6); all of these factors may initiate apoptosis (Harjai et al., 2013). Recently, regulation of the apoptotic process has been considered as a new therapeutic strategy for the treatment of endotoxemia and its complications (Eduardo and Fresno, 2013). Therefore, an in-depth understanding of the molecular regulation of endotoxin-induced apoptosis could provide a scientific basis for the intervention and treatment of the various diseases mentioned in Figure 1.

## ETX Induces Apoptosis *In Vitro* and *In Vivo*

ETX receptors could be divided into the following categories: scavenger receptor (SR), lipopolysaccharide-binding protein (LBP), cluster of differentiation antigen 14 (CD14) (Ulevitch and Tobias, 1995), β2-integrins (such as CD11/CD18), and Toll-like receptors (TLRs). They are extensively distributed on the surface of immune cells such as monocytes, macrophages, and neutrophils and play an important role in the recognition and elimination of ETX in the host. The binding of LPS to LBP activates transmembrane receptors such as SR, β2-integrins, and TLRs to transmit signals into the cell. These signals further promote the activation of transcription factors and induce the expression of pro-inflammatory genes. The expression of these overproduced pro-inflammatory products can further cause the induction of apoptosis. Apoptosis is induced by the intrinsic (mitochondrial) pathway and the extrinsic (death receptor-mediated) pathway (Danial and Korsmeyer, 2004). The intrinsic pathway involves mitochondria and depends mainly on Bcl2 family proteins and caspase 9/7/6/3 (Hengartner, 2000; Leist and Jäättelä, 2001). The extrinsic pathway is involved in death signaling, such as TNF-α with TNF receptor 1 (TNFR1). Then, the activation of death signaling further activates caspase-8, which cleaves procaspase-3 into its active form and activates various genes such as P53, Fas, Bcl2, and NF-κB alone (Micheau and Jürg, 2003; Peter and Krammer, 2003). In contrast, the NF-κB signaling cascade and p53 activate pro-apoptotic Bcl2 family proteins and inhibit anti-apoptotic Bcl2 family proteins and inhibitors of apoptotic proteins (cIAPs) via transcription, as well as several other p53 targets such as BAX, Noxa (Latin for damage, a BH3 protein involved only in regulating cell death decisions), p53-upregulated apoptosis regulator (PUMA) and BID to trigger apoptosis. Furthermore, p53 also transactivates several other genes that may contribute to apoptosis, including phosphatase and tensin homolog deleted on chromosome-10 (PTEN), APAF1, Perp, and genes that cause elevated levels of reactive oxygen species (ROS). The overproduction of ROS leads to extensive oxidative damage to all components of mitochondria. Mitochondrial DNA damage induced by ROS further disrupts the oxidative phosphorylation of mitochondria, which leads to a range of human diseases. Herbal ingredients are often involved in the treatment of endotoxemia with anti-inflammatory, antioxidant, and anti-apoptosis effects, which are interrelated and affect each other. Interestingly, however, there is no one-to-one correlation between anti-inflammatory/antioxidant and anti-apoptotic/pro-apoptotic.

Endotoxin has been reported to significantly induce apoptosis *in vitro* and *in vivo* (O’Brien and Abraham, 2004; Neff et al., 2006; Li et al., 2018). ETX can directly stimulate the production of cytokines by monocytes-macrophages to activate the body’s local or overall immune system against bacterial infections. On the other hand, if the infection is severe, large amounts of ETX in the body come into contact with mononuclear-macrophages and release vast inflammatory cytokines such as TNF-a leading to an uncontrolled inflammatory response. In addition, ETX stimulates the production of large amounts of nitric oxide (NO) by inducing non-calcium-dependent inducible nitric oxide synthase (INOS) in vascular cells. ETX also activates monocytes, macrophages, and neutrophils, and further promotes the expression of apoptosis-related receptors, such as Fas, TLR-2, and CD14, which can also induce apoptosis and tissue damage (Hotchkiss et al., 2002). Apoptosis of parenchymal cells can lead to dysfunction in multiple organs, while apoptosis of immune cells manifests as impaired host immune tolerance and immunosuppression (Hotchkiss et al., 2005), ultimately leading to septic shock, systemic inflammatory response syndrome (SIRS), organ dysfunction syndrome (MODS), and death. Interestingly, in sepsis, both pro- and anti-apoptotic members of the apoptosis-related pathway are upregulated, but the ratio is more favorable to the pro-apoptosis. In addition, lymphocytes are susceptible to endotoxin-induced apoptosis, with increased apoptosis of CD4^+^ T cells (Jäättelä and Tschopp, 2003), B cells, and follicular dendritic cells in the spleen of patients, while oxidative stress can lead to apoptosis of thymocytes (Fehsel et al., 1995). Although there is evidence that granulocytes can limit inflammatory damage through apoptosis, this is macrophage-dependent because the phagocytosis of apoptotic cells by macrophages effectively prevents the release of toxic content from dead cells. If a large number of macrophages undergo apoptosis, the apoptotic granulocytes will eventually lyse and release toxic molecules such as elastase and myeloperoxidase from neutrophils, major base proteins (MBP) from eosinophils, Eos cationic protein (ECP), and Eos-derived neurotoxin (EDN), leading to further uncontrolled inflammatory responses. However, the non-immune cells are mainly parenchymal cells of the liver, lung, and intestine (Suzuki et al., 2011; Liu H. et al., 2015; Guo et al., 2019), while cardiomyocytes (Liu et al., 2013; Zhang et al., 2015; Tian et al., 2019) and nerve cells (Peña et al., 2011; Reichardt et al., 2017) of apoptosis have also been reported.

## Anti-Apoptosis Mechanism of Herbal Medicine in Endotoxemia

It has been observed that various herbal ingredients can improve the healing of endotoxemia, not only through anti-inflammatory effects, but also through their regulation of apoptosis in many types of immune cells (including lymphocytes and macrophages), endothelial cells, and parenchymal cells in different solid organs, especially heart, liver, and lung (Cheng et al., 2014). Although apoptosis has been extensively studied in various diseases such as cancer, neurodegenerative diseases, and HIV infection, its role in sepsis and its regulation as a novel therapeutic approach in the treatment of endotoxemia has attracted attention in recent years.

### Sanhuang Xiexin Decoction and Its Metabolites

**FIGURE 2:**
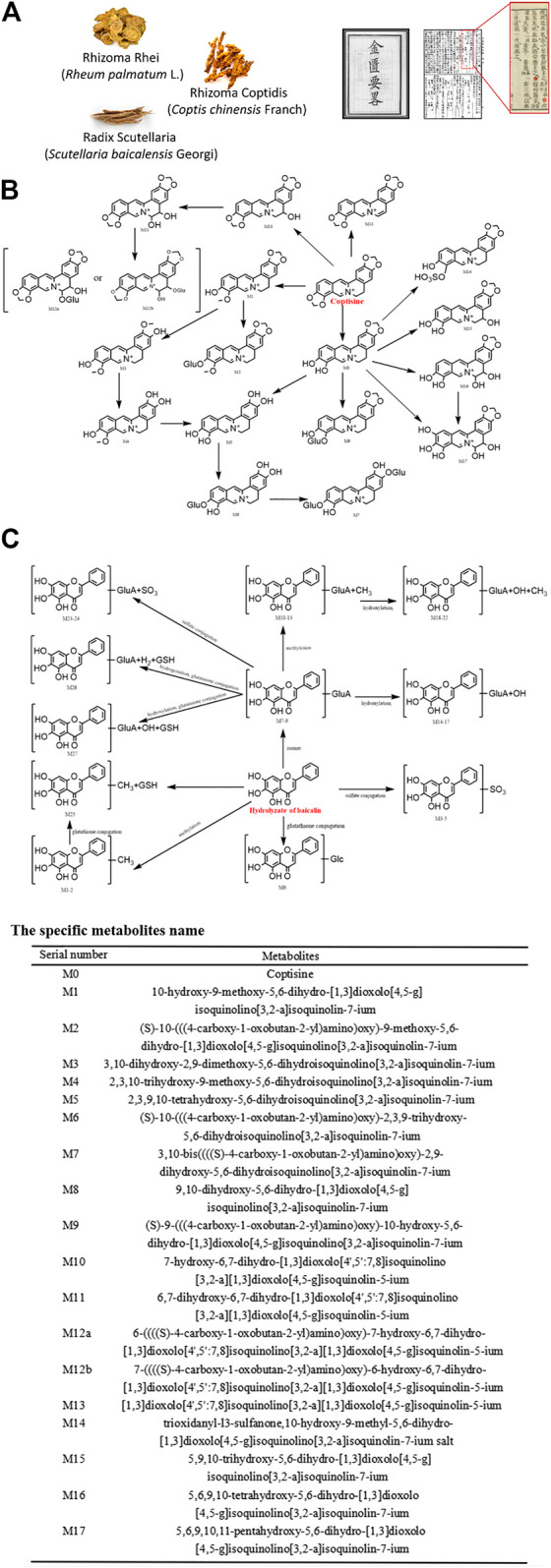
Metabolic pathways of main components of Sanhuang Xiexin Decoction.

Sanhuang Xiexin decoction was firstly described in the *Synopsis of Golden Chamber* by Zhongjing Zhang of the Eastern Han Dynasty and is a classical formula widely used in Chinese medicine to treat fire and detoxify. It is composed of Rhizoma Rhei (*Rheum palmatum* L.), Rhizoma Coptidis (*Coptis chinensis* Franch), and Radix Scutellaria (*Scutellaria baicalensis* Georgi). Previous studies have shown that Sanhuang Xiexin Decoction has effective therapeutic effects on endotoxemia through anti-inflammation (Lo et al., 2005a; Lo et al., 2005b). Other recent studies have shown that it also possesses potent anti-apoptotic activity. The main anti-apoptotic ingredients are aloe-emodin, coptisine, and baicalin. Figure 2 depicts its formula and the metabolic steps and products of coptisine and baicalin *in vivo*. For example, aloe-emodin can attenuate myocardial infarction and cardiomyocyte apoptosis (Yu et al., 2019). The metabolites are rhein, aloe-emodin sulfates/glucuronides, and rhein sulfates/glucuronides (Yu et al., 2016). Among them, rhein exhibited significant anti-apoptosis in endotoxemia and showed significant protective effects against endotoxin-induced kidney injury (Yu et al., 2015). Coptisine, a major compound from Rhizoma Coptidis that clears heat and dampness, purging fire for removing the toxin has been reported to exhibit protective effects on HaCaT keratinocytes by blocking the mitochondria-dependent apoptotic pathway (Choi, 2019). Furthermore, baicalin is the most reported anti-apoptotic component of Sanhuang Xiexin Decoction, showing anti-apoptotic effects in a variety of cell types, including cardiomyocytes (Liou et al., 2011; Liou et al., 2012), C2C12 myoblast (Pan et al., 2019), arterial endothelial cells (Shou et al., 2017), neuronal cells (Zheng, et al., 2015), hepatic stellate cells (Wu et al., 2018a; Wu et al., 2018b), endplate chondrocytes (Pan et al., 2017), renal cells (Zhu et al., 2016) and colonic epithelial cells (Yao et al., 2016). Baicalein 6-*O-β-* *D*-glucopyranuronoside (Akao et al., 2013) and baicalein (Wang et al., 2015) are the main metabolites of baicalin that inhibit the apoptosis of intestinal epithelial cells, thus reducing the chance of endotoxemia in cirrhosis (Liu Y. et al., 2015) and cardiomyocyte injury (Zhu et al., 2019).

### Molecular Docking Prediction of Anisodamine for Anti-Apoptotic Treatment of Endotoxemia

**FIGURE 3:**
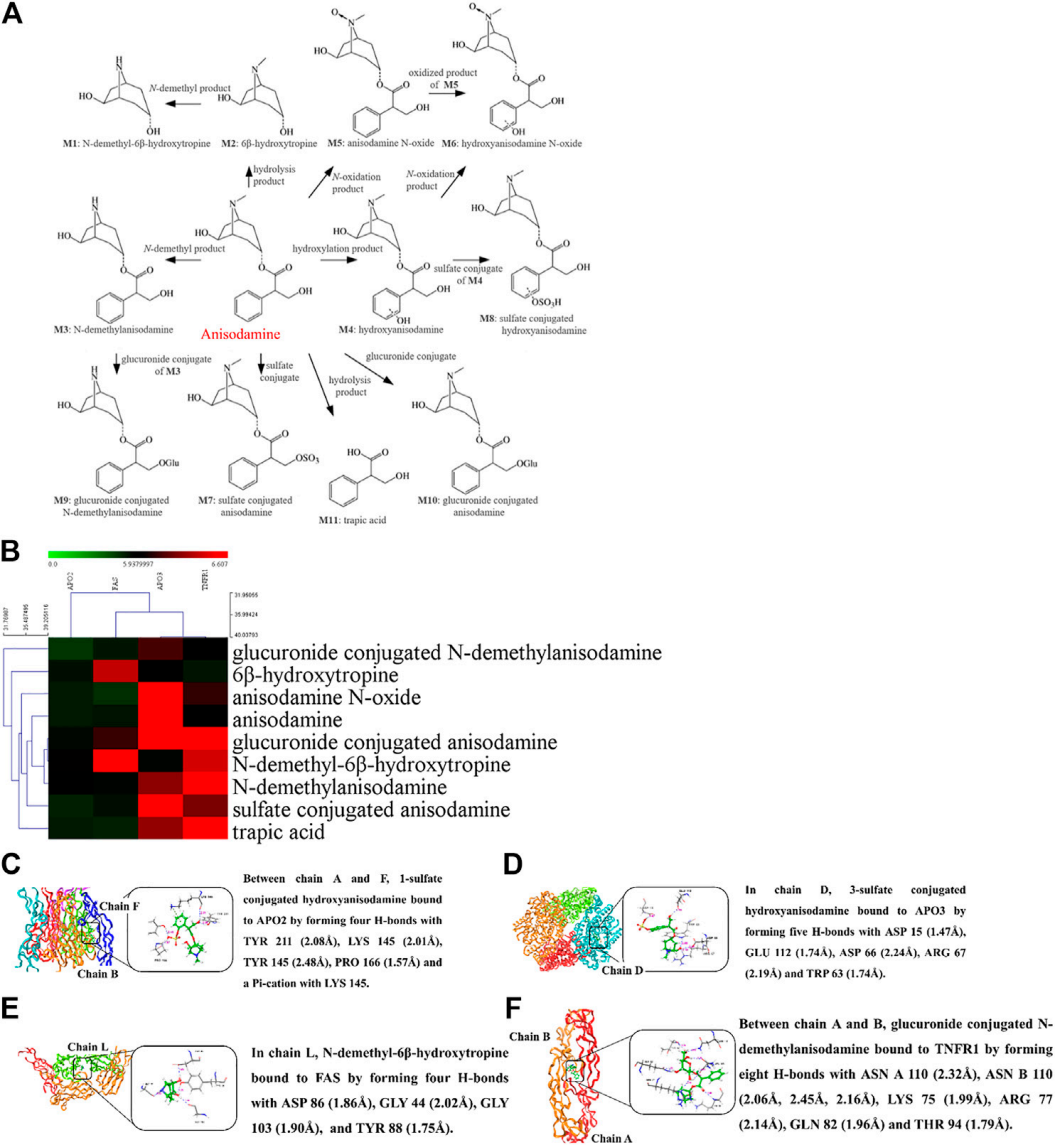
Molecular docking of anisodamine binding with apoptosis receptor of endothelial cells.

Anisodamine is a well-known natural product that is widely used to improve the survival of patients with septic shock. The binding capacity of anisodamine and its 23 intracorporal metabolites (Chen et al., 2005) to endothelial cell apoptosis-related receptors was evaluated by molecular docking technology. As shown in Figure 3, anisodamine and its metabolites bind mainly to TNFR1 and APO3 with high affinity. Other individual metabolites, such as N-demethyl-6 beta hydroxytropine and 1-sulfate conjugated hydroxyanisodamine also bind to FAS and APO2 with strong affinity. Thus, the therapeutic effect of anisodamine on endotoxemia involves molecular mechanisms associated with apoptosis. However, further evaluation regarding the molecular mechanisms of anisodamine *in vitro* and *in vivo* is necessary.

### Other Anti-Apoptosis Ingredients Derived From Herbal Medicines

**FIGURE 4:**
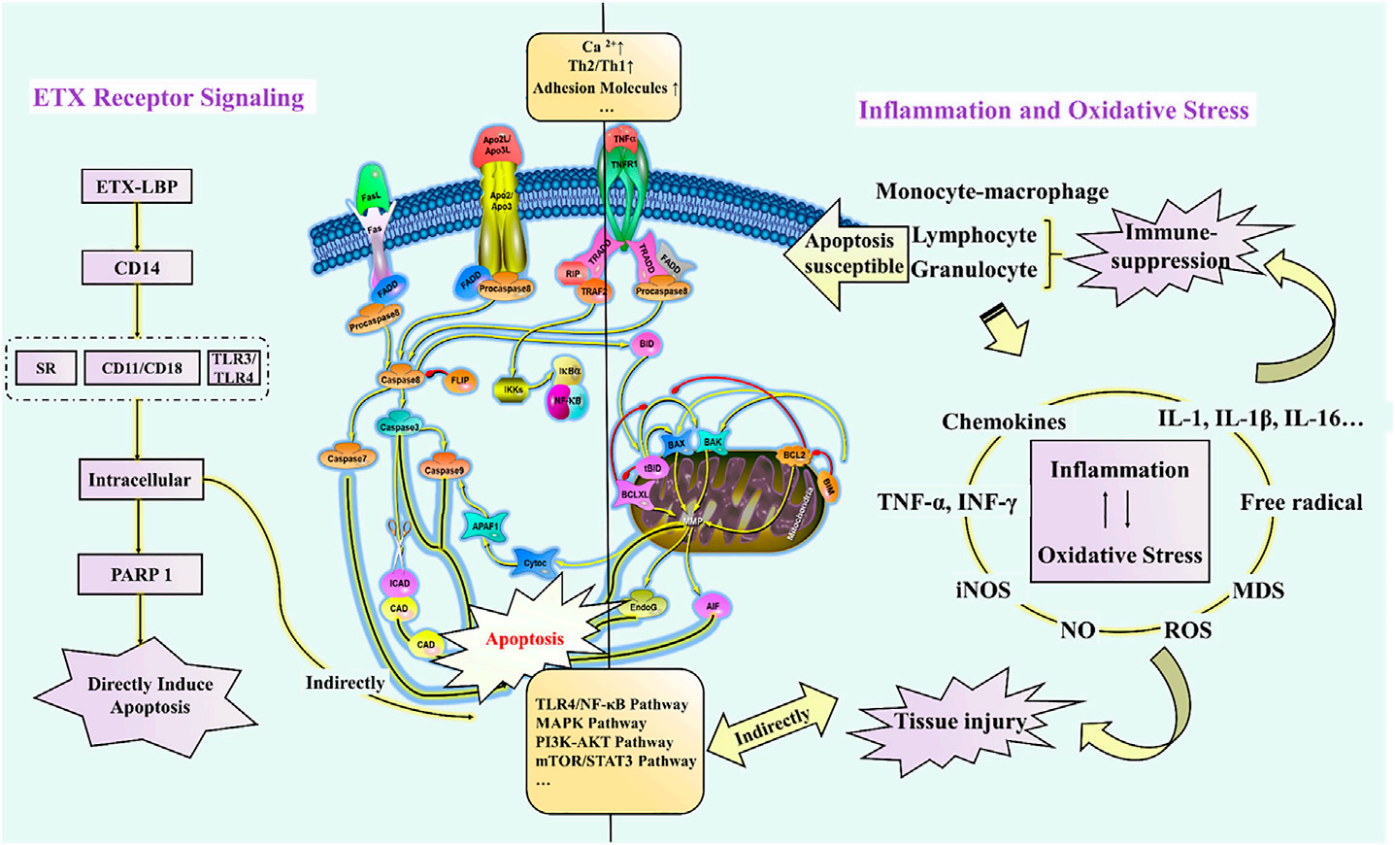
Schematic diagram of the molecular mechanism of endotoxin-induced apoptosis.

As seen in Figure 4, immune paralysis and organ damage are the main culprits for the poor prognosis of endotoxemia. Many herbal ingredients are effective in the treatment of endotoxemia by inhibiting apoptosis. Their effects, target organs/cells, dosage, relevant targets, and indications have been summarized in Table 1. The activation of the TLR4/NF-κB pathway leads to exacerbation of inflammation and apoptosis, which in turn leads to worsening endotoxemia. Therefore, inhibition of inflammation, oxidative stress, and apoptosis is a new research focus in the treatment of endotoxemia (Guan et al., 2019; Li et al., 2019; Zhao et al., 2020).

**TABLE 1:** Effect of herbal ingredients on anti-apoptosis related pathway molecular (excepted those mentioned in the text).

Herbal medicine	Effects	Related targets	Endotoxemia related indications	References
Punicalagin (PNG)	Anti-apoptosis, nephroprotection, anti-inflammation, anti-oxidant	↓: Serum Cr, NGAL, KIM-1, IL-18, TNF-α, IL-6; MDA, NO, MPO, iNOS. Bax/Bcl2, caspase-3/-8/-9	Sepsis complication of acute kidney injury	Fouad et al. (2016)
Thaliporphine	Anti-inflammation, anti-apoptosis; radioprotection	↓: p38/NF-κB pathway; TNF-a, caspase 3, serum cTnI, LDH, Reverse steeper EDPVR, Gentler ESPVR	Cardiac depression	Lee et al. (2010)
		↑: PI3K/Akt/mTOR pathway		
Apocynin	Hepatocyte protection, anti-apoptosis, anti-oxidation	↓: Hydroperoxides, MDA, SOD, NADPH, POX	Endotoxemia	Ben et al. (2000)
		↑: CAT, SOD, G-POX		
Stevioside	Anti-apoptosis, anti-oxidation, anti-inflammation, immunoregulation	↓: SOD, CAT, GSH, MDA, NO, DPPH; TNF-α, IL-1β, IL-6	Acute liver injury	Latha et al. (2017)
		↑: AST, ALT		
Soybean oil, olive oil	Anti-inflammation, immunoregulation	↓: Mitochondrial pathway, Apoptosis of lymphocyte, Caspase-8, Bax, Bcl-xl, caspase-3, TNF-α, IL-6	Sepsis complications of acute lung injury and acute respiratory distress syndrome	Bi et al. (2010)
		↑: PI3K/Akt phosphorylation		
Cordyceps sinensis	Anti-apoptosis, anti-inflammation, immunoregulation	↓: NF-κB, TNF-α, AST, NO, AST, caspase-3/-6, PARP	Sepsisshock complication of hepaticdysfunction	Chen and Wu. (2014)
		↑: Phosphorylation of MAPK (ERK1/2), SOD, IL-10		
Garlic	Anti-inflammation, pro-apoptosis, immunoregulation, anti-platelet, anti-hypertension, antioxidation	↓: TNF-a, IL-6	Preeclampsia	Makris et al. (2005)
		↑: IL-10, sTRAIL/Apo-2L		
Salvia miltiorrhizae	Improve microcirculation, anti-inflammation, antioxidation, anti-apoptosis, mucosal protection, macrophage protection	↓: p65NF-κB, PLA2, Bax	Severe acute pancreatitis; obstructive jaundice	Zhang et al. (2009)
Sophocarpine	Anti-inflammation, anti-apoptosis, antioxidation	↓: p38/JNK, NF-κB, PI3K/AKT, Bcl-xl, H_2_O_2_, O_2_, NO, CYP2E/NRF2, CYPE2, ROS, ALT, ALT, ALP, AST, IL-1β, TNF-α, IL-6, Cyto-C, Apaf1, caspase-9/-3	Septicliver injury	Zheng et al. (2018)
		↑: SOD, CAT, GSH, SOD1, Nrf2		
Astragalusroot	Cytoprotection, anti-apoptosis, immunoregulation	↓: ROS, caspase-3, p53, mitochondrial membrane depolarization	Endotoxemia	Wu et al. (2019)
		↑: Bcl2		
Astragalin	Antioxidation, anti-apoptosis, anti-inflammation, antianaphylaxis	↓: NF-κB, MAPK, TLR4, JNK pathways; eotaxin-1, ROS, caspase-3, NADPH, PLCγ1-PKCβ2-NADPH	Allergic airway diseases; peribronchial eosinophilia	Cho et al. (2014)
		↑: Akt, ERK		
Matrine	Anti-inflammation, antioxidation, anti-fibrosis, antiviral	↓: NF-κB, MIP-2, MPO, MDA, TNF- α, IL-6, IL-8, ALT, AST, LDH, ALP, NO, sICAM-1, caspase-3	Acute liver injury, Hepaticischemia	Zhang et al. (2011)
Green teapolyphenols, catechins, flavandiols, flavanols, phenolicacids	Anti-apoptosis, geno protective, antioxidation	↓: AST, ALT, TB, ALB, NO	Liver damage	Allam et al. (2017)
		↑: TAC		
Siniinjection	Anti-apoptosis	↓: MABP, iNOS, PI, IL-6, IL-18, ET-1, NO	Septic shock	Pei et al. (2013)
12-O-Tetradecanoyl-phorbol 13-Acetate (TPA)	Anti-apoptosis	↓: NF-κB	Endotoxemia	Sunil et al. (2002)
		↑: C/EBP, p38 MAPK		
Strawberry	Anti-inflammation, antioxidation, anti-apoptosis	↓: NF-κB, NO, iNOS, CAT, SOD, ROS, Caspase 3, PiκBα, TNF-α, IL-1β, IL-6	Human dermalfibroblast	Gasparrin et al. (2018)
		↑: pAMPK, SIRT1463, PGC1α, GPX, GR, GST, IL-10, Nrf2-AMPK	Inflammationliver injury	
Betulinicacidderivative BA5	Immunoregulation	↓: NF-κB, TNF-α, IL-2, IL-4, IL-6, IL-17A, IFN-γ, NO, Activated LY, CD4^+^ Tcellcalcineurin activity	Delayed hypersensitivity	Meira et al. (2017)
Garlic oildiallyldisulfide diallyl trisulfide	Anti-inflammation, antioxidation, anti-apoptosis	↓: nitrate/nitrite	Intestinaldamage	Chiang et al. (2006)
Rubiaceae-typecyclo-peptide (RA-V)	Anti-inflammation	↓: NF-κB, NO, IL-8, IL-6, MCP-1, P-p65, P-IκBα	Septic shock	Wang et al. (2018)
		↓: PI3K/AKT, TAK1 + TAB2		
Cinnamaldehyde (CA) Linalool (LIN)	Anti-inflammation	↓: NF-κB, TLR4/MD2, MyD88, NLRP3, ASC, caspase-1, TNF-α, IL-1β, IL-18, nitrate/nitrite, IFN-γ, HMGB-1	Endotoxemia	Lee et al. (2018)
Gryllusbimaculatus Extracts (GBE)	Anti-apoptosis, anti-inflammation, antioxidation	↓: ROS, NO, IL-6, TNF-α, TG, TLR4, P-JNK, P-p38, OHdG, p-MLCK, p-ROCK, p-srcFK	Alcoholic liver diseases	Hwang et al. (2019)
			Intestinal inflammation	
Scytonemin	Antiproliferative	↓: cdc25C, p-cdc25C, Cell cycleregulatory kinases, GST-PLK, GST-Tie2, Protein kinase A, Protein kinase Cβ2, CDK1/cyclin B, GST-Myt1, GST-checkpoint kinase 1, GST-polo-like kinase 1	Endotoxemia	Stevensonet al. (2002)

Meng et al. confirmed that sanguinarine attenuates ETX-induced inflammation and apoptosis by blocking the TLR4/NF-κB pathway (Meng et al., 2018). However, in general, the mechanism of herbal ingredients based on this pathway for the treatment of endotoxemia is less studied, and it remains to be verified whether the inhibition of this pathway signal can be used to evaluate and screen potential herbal ingredients. Furthermore, leukocyte adhesion molecules have been suggested as new targets for the development of anti-inflammatory agents (Mukaida et al., 1996), which is mainly attributed to the enhancement of TNF-α-induced apoptosis by β2 integrins (CD11/CD18) at 4 and 8 h after ETX stimulation (Walzog et al., 1997). Moreover, free total rhubarb anthraquinones (FTRAs) attenuated intestinal injury and enhanced intestinal barrier function by regulating intestinal immune function in rats with endotoxin-induced acute pancreatitis (Xiong, et al., 2018). In addition, endotoxemia can also induce apoptosis through oxidative stress. For example, piperlongumine exhibits prominent anti-inflammatory effects by inhibiting the ETX-induced p65 NF-κB signaling cascade and markedly suppresses cytokine storm production, which is closely associated with ROS-mediated induction of late apoptosis and reduced expression of anti-apoptotic Bcl2 protein (Thatikonda et al., 2020). To sum up, Figure 4 reveals how the recognition signals of endotoxin, inflammation, and oxidative stress responses induce apoptosis directly or indirectly. These herb ingredients show significant therapeutic effects on endotoxin, inhibiting endotoxin-induced elevation in Ca^2+^ and adhesion factors, and activation of signaling pathways such as TLR4/NF-κB, MAPK, PI3K-AKT, and mTOR/STAT3.

## Future Prospects

In conclusion, numerous studies have shown that the anti-apoptotic treatment of endotoxemia can improve immunosuppression and protect parenchymal organ function. A range of bacterial components such as ETX can induce a cellular stress response and induce apoptosis. However, ETX, as a caspase inhibitor, can inhibit apoptosis by engaging its O-antigen (O Ag) moiety to directly bind caspases, contributing to the successful colonization of bacteria to support their intracellular propagation (Günther et al., 2019), but to the detriment of humans. In addition, it is interesting to note that many anti-apoptotic herbal ingredients in endotoxemia exhibit pro-apoptotic activity in cancer. For example, salidroside increases survival in endotoxemic mice (Guan et al., 2011) and has been reported to inhibit apoptosis in bone marrow mesenchymal stem cells (Wei et al., 2013), H9c2 cardiomyocytes (Sun et al., 2018), pulmonary arterial smooth muscle cells (Gui et al., 2017), pheochromocytoma cells (Hu et al., 2016), neural stem cells (Yan et al., 2018), cardiomyocytes (Zhu et al., 2015) and umbilical vein endothelial cells (Chen and Wu, 2014), while inducing apoptosis in colorectal cancer cells (Fan et al., 2016) gastric cancer AGS cells (Rong et al., 2020). It is thus clear that anti-apoptosis is not a single effective approach for the treatment of endotoxemia with herbal medicine, but must be combined with anti-inflammation, anti-oxidative stress, and anticoagulation to improve the prognosis of patients with endotoxemia. In addition, it should be noted that the anti-apoptotic activity of herb ingredients is also affected by the pathological state of the host. In any case, anti-apoptotic therapy plays an important role in endotoxemia but has long been neglected, and a considerable number of experimental studies on endotoxemia do not involve the detection of apoptotic-related indicators. This suggests that the impact of apoptosis on the pathophysiological process of endotoxemia should be emphasized and that anti-apoptosis is a potential new approach for the treatment of endotoxemia.

## Abbreviations

APAF1, Apoptotic protease activating factor-1; BAK1, B-Cell leukemia-2 antagonist killer-1; BAX, Bcl2 associated X-protein; Bcl2, B-Cell leukemia-2; BID, BH3 interacting death domain; BIM, B-Cell leukemia-2 interacting protein; CLSM, Confocal laser scanning microscopy; ECP, Eos cationic protein; EDN, Eos-derived neurotoxin; ETX, endotoxin; FADD, Fas-associated via death domain; FasL, Fas ligand; FM, Fluorescence microscopy; ICAD, Inhibitor of caspase-activated dNase; INOS, inducible nitric oxide synthase; LPS, lipopolysaccharide; MBP, Major base proteins of eosinophils; MODS, organ dysfunction syndrome; MOMP, Mitochondrial outer membrane permeabilization; NO, nitric oxide; OM, Optical microscopy; PTEN, phosphatase and tensin homolog deleted on chromosome-10; PUMA, p53-upregulated modulator of apoptosis; p53AIP1, p53-regulated apoptosis-inducing protein-1; RIP, Receptor-interacting protein; ROS, reactive oxygen species; SIRS, systemic inflammatory response syndrome; TNFR1, TNF receptor 1; TLM, Transmission electron microscopy; TRADD, Tumor necrosis factor receptor-1-associated death domain
